# Supplementary material for: Relative Validity of the Groningen IBD Nutritional Questionnaire (GINQ-FFQ): A Food Frequency Questionnaire Designed to Assess Nutritional Intake in Patients with Inflammatory Bowel Disease
Source: Nutrients. 2025 Jan 10;17(2):239. doi: 10.3390/nu17020239 (PMC11768067; doi:10.3390/nu17020239)
Supplement: Supplementary file 1 [file nutrients-17-00239-s001.zip › Supplement S1.pdf]

Patient identification number

Food diary version 1.0

Food Diary

Why a food diary?

This food diary can be used to write down what you eat and drink daily. This way, the researcher can know what food u normally consume and how much. We want to use this information to determine your mean food intake. You are supposed to fill out this food diary on 1 weekend-day and 2 regular weekdays (3 days in total). Please write everything down you eat and/or drink during the day as precisely as possible, also the in-between snacks. Filling out the diary needs to be done as precisely as possible. Therefore, we advise you to first read all guidelines before you start.

Food diary version 1.0

Guidelines for filling out the food diary:

- Write down your food intake for 3 days, with one of the 3 days being a Saturday or a Sunday. The days may not follow each other, so e.g. not Mon-Tue or Wed-Thu in the same week.
- On the next page you will find a schedule to keep track of which days you recorded your food intake.
- Note down exactly what you eat and drink, every little bit counts. Also note small snacks such as liquorice/sweets. Write down exactly what you eat, not the amount you prepare or have served.
- It is best to weigh everything. The quantities can also be given in household measurements, such as cups, mugs, slices, bowls. Sometimes the weight can also be derived from the packaging. Since one bowl is not the same as another, you can see examples on the next page and fill in your measurements in the provided table. Indicate the quantity and type of food with the brand name for everything you eat and drink.
- Describe (if applicable) the specific recipe for a prepared meal with the quantities. If you only eat a portion, mention this.
- All details count:
    - Do you have sugar/milk in your tea or coffee?
    - What kind of frying fat or butter do you use?
    - Have you written down everything you drink? Lean or semi-skimmed?
    - Have you used extra salt in your meal?
- Some people like to keep the booklet with them. That way they don't forget to write anything down. If necessary, use a small notepad so that you can add to the food diary at a later time.
- For hot meals, weigh the quantities as much as possible, or copy the weight from the packaging. For meat, rice and pasta, note the weight before preparing.
- When you cook for multiple people, it is difficult to remember how much you have had, everything is mixed up. You can write down the recipe, and then write down how much you have eaten (e.g. half, a quarter ...)
- Provide as much detail as possible about food products, the packaging can help you write everything down properly. For example, for ‘cheese’: one whole pre-cut slice of Beemster 30+ cheese, matured. And also, if you use the ‘less salt’ variant of something.

Remember that you can never write too much. Good luck filling out the diary!

Food diary version 1.0
Household measurements

You can measure your standard dishes with a measuring cup. For other foods, it is best to weigh them, or infer from the packaging.

When indicating quantities, you may use household measurements. However, because these capacity measurements are not the same for everyone, we ask you to measure the household measurements used with a measuring cup. For example, you fill a mug with water as you normally would with milk and pour this into the measuring cup. You can then read the quantity and note it down below.

**Household measurement**	**Measurement**
1 glass	
1 large glass	
1 teacup	
1 coffee cup	
1 mug	
1 dessert bowl	
1 soup bowl	
1 deep plate	
1 wine glass	
1 beer glass	
…………………………………………………………	

Below we have listed the standard contents of a cup, glass, mug, bowl, soup cup and soup plate:

- 1 cup = 125 ml
- 1 glass = 150 ml
- 1 mug = 225 ml
- 1 bowl = 200 ml
- 1 soup bowl = 250 ml
- 1 deep plate = 200 ml

Patient identification number

Food diary version 1.0

thinly spread	average	thickly spread

thinly spread

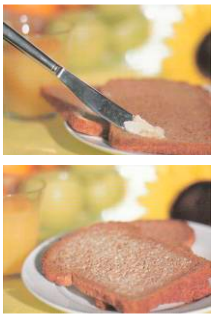

average

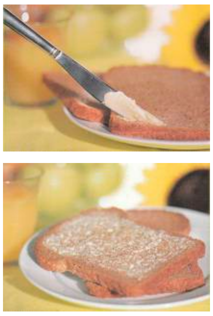

thickly spread

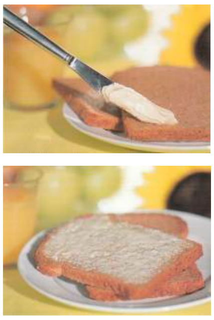

✓

Please indicate below how you drink your coffee and tea:

- Tea without / with* sugar: ………. teaspoons / cubes* sugar
- Coffee without / with* sugar, ………. teaspoons / lumps* of sugar

Coffee black / with semi-skimmed coffee milk / with full-fat coffee milk / with coffee cream / with coffee milk powder*

Please complete the following:

We would like to know on which days you have recorded your food intake. An example of a completed schedule is:

**Record day**	**Day**	**Date**
remarks	*2 x Mon, Tue, Wed, Thu of Fri*<br>*1x Sat of Sun*	*Non consecutive days (so not Mon-Tue or Wed-Thu in the same week)*
1	Tuesday	October 13
2	Thursday	October 15
3	Sunday	October 18

In the table below, please fill out the day and date of the 3 days on which you recorded your food intake.

**Record day**	**Day**	**Date**
remarks	*2 x Mon, Tue, Wed, Thu of Fri*<br>*1x Sat of Sun*	*Non consecutive days (so not Mon-Tue or Wed-Thu in the same week)*
1		
2		
3		

Patient identification number

Food diary version 1.0

Day 1: Breakfast date:……………….. ………..day

Quantity	Food item
	**Bread:**
__________	Slice(s) white/brown/wholemeal bread
__________	Slice(s) rye bread
__________	Slice(s) currant bread
__________	Slice(s) sugar bread
__________	Piece(s) rolls
__________	Piece(s) soft buns
__________	Piece(s) croissant
__________	Piece(s) Other, namely…………………………………………………………………
	**Spread / topped with:**
__________	Butter
__________	Margarine, brand and type,………………………………………………………………
__________	Half-fat margarine, brand and type,………………………………………………....
	Cheese, type……………………………………………………………………………
__________	Cold cuts, type………………………………………………………………………...
__________	Sweet topping, type……………………………………………………………………..
__________	Other, namely ……………………………………………………………………......
__________	
	**Yogurt**
__________	Bowl of low-fat/semi-skimmed/full-fat yoghurt
__________	**Custard**
__________	Bowl of low-fat/semi-skimmed/full-fat custard
	**Quark**
__________	Bowl of low-fat/semi-skimmed/full-fat quark
	**Porridge**
__________	Bowl of porridge
	**The yoghurt, custard, quark filled with:**
__________	Cornflakes
__________	Muesli
__________	Cruesli
__________	Fruit, namely …………………………………………………………..…………………
__________	Other, namely …………………………………………………………………………

Patient identification number

Food diary version 1.0

___________	**Fruit**<br>Pieces of fruit, namely …………………………………………………………………
 ___________<br>___________<br>___________<br>___________<br>___________<br>___________<br>___________<br>___________<br>___________<br>___________	**Drinks:**<br>Water cup/mug/glass*<br>Teacup/mug/glass*<br>Coffee cup/mug/glass*<br>*Skimmed/semi-skimmed/full fat milk cup/mug/glass**<br>Buttermilk cup/mug/glass*<br>*Skimmed/semi-skimmed/full fat chocolate milk cup/mug/glass**<br>*Skimmed/semi-skimmed/full fat drinking yoghurt cup/mug/glass**<br>Fruit juice namely………………………………………..…….cup/mug/glass*<br>Soft drink namely………………………………………………cup/mug/glass*<br>Other, namely …………………………………………………………………
Remarks:	If you ate/drank something that is not listed above, you can enter it here.<br>…………………………………………………………………………………..……<br>……………………………………………………………………………………………<br>……………………………………………………………………………………………<br>……………………………………………………………………………………………<br>……………………………………………………………………………………………<br>……………………………………………………………………………………………<br>……………………………………………………………………………………………

* Cross-out what does not apply

Patient identification number

Food diary version 1.0

Day 1: DURING THE MORNING date:……………….. ………..day

Quantity	Food item
__________	**Drinks:**
__________	Water cup/mug/glass*
__________	Teacup/mug/glass*
__________	Coffee cup/mug/glass*
__________	*Skimmed/semi-skimmed/full fat milk cup/mug/glass**
__________	Buttermilk cup/mug/glass*
__________	*Skimmed/semi-skimmed/full fat chocolate milk cup/mug/glass**
__________	*Skimmed/semi-skimmed/full fat drinking yoghurt cup/mug/glass**
__________	Fruit juice namely…………………………………………..…….cup/mug/glass*
__________	Soft drink namely……………………………………………….cup/mug/glass*
__________	Other, namely ………………………………………………………………..
	**Cakes and confectionary**
__________	Slice(s) of large cookie/cake type
__________	Cookie(s)/Biscuit(s)
__________	Pastry, type……………………………………………………….…………..........
__________	Candy bar mars / nuts / other……………………………………….……...........
__________	Piece(s) of chocolate milk / dark / white*
__________	Sweets, type…………………………………………………………………………
	**Savoury:**
__________	Cubes of cheese, type…………………………………………………………….
__________	Piece of sausage, type……………………………………………………………..
__________	Toast with …………………………………………………………….................
__________	Handful(s) of chips/nuts
__________	Other, namely …………………………………………………………………….
	**Fruit**
__________	Pieces of fruit, namely ……………………………………………………………...
Remarks:	If you ate/drank something that is not listed above, you can enter it here.<br>……………………………………………………………………………..………<br>……………………………………………………………………………………<br>……………………………………………………………………………………<br>……………………………………………………………………………………<br>……………………………………………………………………………………<br>……………………………………………………………………………………<br>…………………………………………………………………………………

* Cross-out what does not apply

Patient identification number

Food diary version 1.0
<u>Day 1: Bread meal</u> date:……………….. ………..day

Quantity	Food item
	**Bread:**
__________	Slice(s) white/brown/wholemeal bread
__________	Slice(s) rye bread
__________	Slice(s) currant bread
__________	Slice(s) sugar bread
__________	Piece(s) rolls
__________	Piece(s) soft buns
__________	Piece(s) croissant
__________	Piece(s) Other, namely ………………………………………………………..
	**Spread / topped with:**
__________	Butter
__________	Margarine, brand and type,……………………………………………………
__________	Half-fat margarine, brand and type,…………………………………………….
	Cheese,
__________	type…………………………………………………………………………..
__________	Cold cuts, type………………………………………………………………..
__________	Sweet topping, type……………………………………………………………
__________	Other, namely …………………………………………………………….....
	**Drinks:**
__________	Water cup/mug/glass*
__________	Teacup/mug/glass*
__________	Coffee cup/mug/glass*
__________	*Skimmed/semi-skimmed/full fat milk cup/mug/glass**
__________	Buttermilk cup/mug/glass*
__________	*Skimmed/semi-skimmed/full fat chocolate milk cup/mug/glass**
__________	*Skimmed/semi-skimmed/full fat drinking yoghurt cup/mug/glass**
__________	Fruit juice namely…………………………………………..…….cup/mug/glass*
__________	Soft drink namely……………………………………………….cup/mug/glass*
__________	Other, namely ………………………………………………………………
	**Yogurt**
__________	Bowl of low-fat/semi-skimmed/full-fat yoghurt
	**Custard**
__________	Bowl of low-fat/semi-skimmed/full-fat custard
	**Quark**

Patient identification number

Food diary version 1.0

__________	Bowl of low-fat/semi-skimmed/full-fat quark
	**Porridge**
__________	Bowl of porridge
	**The yoghurt, custard, quark filled with:**
__________	Cornflakes
__________	Muesli
__________	Cruesli
__________	Fruit, namely …………………………………………………………..…………………
__________	Other, namely ………………………………………………………………………
	**Fruit**
__________	Pieces of fruit, namely ………………………………………………………….
Remarks:	If you ate/drank something that is not listed above, you can enter it here.<br>………………………………………………………………………………..……<br>……………………………………………………………………………………<br>……………………………………………………………………………………<br>……………………………………………………………………………………<br>……………………………………………………………………………………<br>……………………………………………………………………………………<br>……………………………………………………………………………………<br>……………………………………………………………………………………

* Cross-out what does not apply

Patient identification number

Food diary version 1.0
Day 1: During the day date:……………….. ………..day

Quantity	Food item
	**Drinks:**
__________	Water cup/mug/glass*
__________	Teacup/mug/glass*
__________	Coffee cup/mug/glass*
__________	*Skimmed/semi-skimmed/full fat milk cup/mug/glass**
__________	Buttermilk cup/mug/glass*
__________	*Skimmed/semi-skimmed/full fat chocolate milk cup/mug/glass**
__________	*Skimmed/semi-skimmed/full fat drinking yoghurt cup/mug/glass**
__________	Beer glass/can/bottle*
__________	Glass of wine
__________	Fruit juice namely……………………………………….....……cup/mug/glass*
__________	Soft drink namely……………………………………………….cup/mug/glass*
__________	Other, namely …………………………………………………………………
	**Cakes and confectionary**
__________	Slice(s) of large cookie/cake type
__________	Cookie(s)/Biscuit(s)
__________	Pastry, type………………………………………………………….………….….....
__________	Candy bar mars / nuts / other……………………………………….……...
__________	Piece(s) of chocolate milk / dark / white*
__________	Sweets, type……………………………………………………………………
	**Savoury:**
__________	Cubes of cheese, type……………………………………………………………
__________	Piece of sausage, type…………………………………………………………..
__________	Toast with …………………………………………………………….................
__________	Handful(s) of chips/nuts
__________	Other, namely ………………………………………………………………
	**Fruit**
__________	Pieces of fruit, namely ………………………………………………………..
Remarks:	If you ate/drank something that is not listed above, you can enter it here.
	……………………………………………………………………………...……
	…………………………………………………………………………………
	…………………………………………………………………………………
	…………………………………………………………………………………
	…………………………………………………………………………………

* Cross-out what does not apply

Patient identification number

Food diary version 1.0

<u>Day 1: HOT MEAL</u> date:……………….. ………..day

Quantity	Food item
	**Soup:**
___________	<u>*Plate / cup* soup, packet / can / homemade**</u>
	What is in it?
	……………………………………………………………………………....................
	....
	**Vegetables / Salad:**
___________	Serving spoons vegetables, type………..……………………………………….…...
___________	Tablespoons applesauce
___________	Serving spoons raw vegetables, type…………………………………………...….
	With <u>sauce / dressing</u>* type……......…...……………………………....................
	………………………………………………………………………………………..
	**Meat / meat substitutes:**
___________	Gram beef, type……….……………………………………………………………….
___________	Gram pork, type…...…………………………………………………………………..
___________	Gram poultry, type….…………………………………………………………………
___________	Gram fish, type………………………………………………………………………..
___________	Gram meat substitute, type……………………………...…………………
___________	Gram other, namely, …………………………………………………………….........
___________	Tablespoons *frying butter / olive oil / sunflower oil**
	**Potatoes / rice and pasta:**
___________	Piece of potato (the size of 1 egg) boiled / fried*
___________	Serving spoons fries
___________	Serving spoons cooked rice, noodles
___________	Serving spoons pasta (e.g. pasta, macaroni, noodles),
___________	Other, namely…………………………………………………………………………..
	**Sauce:**
___________	Gravy spoons gravy, made from butter / margarine / cooking and frying fat *
___________	Tablespoons sauce, type..
	**One-pot meal:**<br>If you ate rice or pasta as a one-pot meal today (e.g. nasi, bami, macaroni dish, stew), how did you prepare it?<br>……………………………………………………………………………..………<br>…………………………………………………………………………….…………<br>…………………………………………………………………………………………

rijksuniversiteit groningen

umcg

Patient identification number

Food diary version 1.0

	**Dessert:**
__________	Dish of low-fat/semi-skimmed/full-fat yoghurt
__________	Dish of low-fat/semi-skimmed/full-fat custard
__________	Dish of low-fat/semi-skimmed/full-fat quark
__________	Dish of pudding Ball/piece* of ice cream, type………………………………….....
__________	Piece of fruit, type…………………………………………………………………
__________	Block of cheese, type........................………………………………………..
__________	Other, namely…………………………………………………………...........
	**Drinks:**
__________	Water cup/mug/glass*
__________	Teacup/mug/glass*
__________	Coffee cup/mug/glass*
__________	*Skimmed/semi-skimmed/full fat milk cup/mug/glass**
__________	Buttermilk cup/mug/glass*
__________	*Skimmed/semi-skimmed/full fat chocolate milk cup/mug/glass**
__________	*Skimmed/semi-skimmed/full fat drinking yoghurt cup/mug/glass**
__________	Beer glass/can/bottle*
__________	Glass of wine
__________	Fruit juice namely……………………………………...…….cup/mug/glass*
__________	Soft drink namely…………………………………………………cup/mug/glass*
__________	Other, namely ……………………………………………………………….
Remarks:	If you ate/drank something that is not listed above, you can enter it here.<br>………………………………………………………………………………..……<br>………………………………………………………………………………………<br>………………………………………………………………………………………<br>………………………………………………………………………………………<br>………………………………………………………………………………………

* Cross-out what does not apply

Patient identification number

Food diary version 1.0

<u>Day 1: During the evening</u> date:……………….. ………..day

Quantity	Food item
	**Drinks:**
__________	Water cup/mug/glass*
__________	Teacup/mug/glass*
__________	Coffee cup/mug/glass*
__________	*Skimmed/semi-skimmed/full fat milk cup/mug/glass**
__________	Buttermilk cup/mug/glass*
__________	*Skimmed/semi-skimmed/full fat chocolate milk cup/mug/glass**
__________	*Skimmed/semi-skimmed/full fat drinking yoghurt cup/mug/glass**
__________	Beer glass/can/bottle*
__________	Glass of wine
__________	Fruit juice namely………………………………………..……cup/mug/glass*
__________	Soft drink namely………………………………………………cup/mug/glass*
__________	Other, namely ………………………………………………………………..
	**Cakes and confectionary**
__________	Slice(s) of large cookie/cake type
__________	Cookie(s)/Biscuit(s)
__________	Pastry, type……………………………………………………….…………….....
__________	Candy bar mars / nuts / other……………………………………..…….......
__________	Piece(s) of chocolate milk / dark / white*
__________	Sweets, type…………………………………………………………………….
	**Savoury:**
__________	Cubes of cheese, type…………………………………………………………
__________	Piece of sausage, type…………………………………………………………….
__________	Toast with …………………………………………………………….................
__________	Handful(s) of chips/nuts
__________	Other, namely …………………………………………………………………
	**Fruit**
__________	Pieces of fruit, namely ………………………………………………………
Remarks:	If you ate/drank something that is not listed above, you can enter it here.<br>……………………………………………………………………………..………<br>……………………………………………………………………………………<br>……………………………………………………………………………………<br>……………………………………………………………………………………<br>……………………………………………………………………………

* Cross-out what does not apply

Patient identification number

Food diary version 1.0
Day 2: Breakfast date:……………….. ………..day

Quantity	Food item
	**Bread:**
__________	Slice(s) white/brown/wholemeal bread
__________	Slice(s) rye bread
__________	Slice(s) currant bread
__________	Slice(s) sugar bread
__________	Piece(s) rolls
__________	Piece(s) soft buns
__________	Piece(s) croissant
__________	Piece(s) Other, namely ………………………………………………………..
	**Spread / topped with:**
__________	Butter
__________	Margarine, brand and type,………………………………………………………
__________	Half-fat margarine, brand and type,……………………………………………
	Cheese, type………………………………………………………………………
__________	Cold cuts, type…………………………………………………………………
__________	Sweet topping, type……………………………………………………………
__________	Other, namely ………………………………………………………………....
__________	
	**Yogurt**
__________	Bowl of low-fat/semi-skimmed/full-fat yoghurt
__________	**Custard**
__________	Bowl of low-fat/semi-skimmed/full-fat custard
	**Quark**
__________	Bowl of low-fat/semi-skimmed/full-fat quark
	**Porridge**
__________	Bowl of porridge
	**The yoghurt, custard, quark filled with:**
__________	Cornflakes
__________	Muesli
__________	Cruesli
__________	Fruit, namely ………………………………………………………..…………………
__________	Other, namely ………………………………………………………………………
	**Fruit**
__________	Pieces of fruit, namely ……………………………………………………………

Patient identification number

Food diary version 1.0

	**Drinks:**
__________	Water cup/mug/glass*
__________	Teacup/mug/glass*
__________	Coffee cup/mug/glass*
__________	*Skimmed/semi-skimmed/full fat milk cup/mug/glass**
__________	Buttermilk cup/mug/glass*
__________	*Skimmed/semi-skimmed/full fat chocolate milk cup/mug/glass**
__________	*Skimmed/semi-skimmed/full fat drinking yoghurt cup/mug/glass**
__________	Fruit juice namely……………………………………..…….cup/mug/glass*
__________	Soft drink namely………………………………………………cup/mug/glass*
__________	Other, namely ………………………………………………………………..
Remarks:	If you ate/drank something that is not listed above, you can enter it here.<br>…………………………………………………………………………………...……<br>…………………………………………………………………………………………<br>…………………………………………………………………………………………<br>…………………………………………………………………………………………<br>…………………………………………………………………………………………<br>…………………………………………………………………………………………<br>…………………………………………………………………………………………

* Cross-out what does not apply

Patient identification number

Food diary version 1.0

Day 2: DURING THE MORNING date:……………….. ………..day

Quantity	Food item
________	**Drinks:**
________	Water cup/mug/glass*
________	Teacup/mug/glass*
________	Coffee cup/mug/glass*
________	*Skimmed/semi-skimmed/full fat milk cup/mug/glass**
________	Buttermilk cup/mug/glass*
________	*Skimmed/semi-skimmed/full fat chocolate milk cup/mug/glass**
________	*Skimmed/semi-skimmed/full fat drinking yoghurt cup/mug/glass**
________	Fruit juice namely……………………………………………..…….cup/mug/glass*
________	Soft drink namely………………………………………………….cup/mug/glass*
________	Other, namely …………………………………………………………………
	**Cakes and confectionary**
________	Slice(s) of large cookie/cake type
________	Cookie(s)/Biscuit(s)
________	Pastry, type………………………………………………………….…………..........
________	Candy bar mars / nuts / other……………………………………………….….……...........
________	Piece(s) of chocolate milk / dark / white*
________	Sweets, type……………………………………………………………………………
	**Savoury:**
________	Cubes of cheese, type………………………………………………………………
________	Piece of sausage, type………………………………………………………………..
________	Toast with ………………………………………………………………..................
________	Handful(s) of chips/nuts
________	Other, namely ………………………………………………………………………
	**Fruit**
________	Pieces of fruit, namely ………………………………………………………………
Remarks:	If you ate/drank something that is not listed above, you can enter it here.<br>……………………………………………………………………………………..………<br>………………………………………………………………………………………<br>………………………………………………………………………………………<br>………………………………………………………………………………………<br>………………………………………………………………………………………<br>………………………………………………………………………………………<br>………………………………………………………………………………………

* Cross-out what does not apply

Patient identification number

Food diary version 1.0

<u>Day 2: Bread meal</u> date:……………….. ………..day

Quantity	Food item
	**Bread:**
__________	Slice(s) white/brown/wholemeal bread
__________	Slice(s) rye bread
__________	Slice(s) currant bread
__________	Slice(s) sugar bread
__________	Piece(s) rolls
__________	Piece(s) soft buns
__________	Piece(s) croissant
__________	Piece(s) Other, namely …………………………………………………………..
	**Spread / topped with:**
__________	Butter
__________	Margarine, brand and type,………………………………………………………
__________	Half-fat margarine, brand and type,………………………………………….
	Cheese,
__________	type………………………………………………………………………………...
__________	Cold cuts, type…………………………………………………………………
__________	Sweet topping, type…………………………………………………………….
__________	Other, namely ……………………………………………………………....
	**Drinks:**
__________	Water cup/mug/glass*
__________	Teacup/mug/glass*
__________	Coffee cup/mug/glass*
__________	*Skimmed/semi-skimmed/full fat milk cup/mug/glass**
__________	Buttermilk cup/mug/glass*
__________	*Skimmed/semi-skimmed/full fat chocolate milk cup/mug/glass**
__________	*Skimmed/semi-skimmed/full fat drinking yoghurt cup/mug/glass**
__________	Fruit juice namely………………………………………..…….cup/mug/glass*
__________	Soft drink namely……………………………………………….cup/mug/glass*
__________	Other, namely ………………………………………………………………..
	**Yogurt**
__________	Bowl of low-fat/semi-skimmed/full-fat yoghurt
	**Custard**
__________	Bowl of low-fat/semi-skimmed/full-fat custard
	**Quark**

Patient identification number

Food diary version 1.0

__________	Bowl of low-fat/semi-skimmed/full-fat quark
	**Porridge**
__________	Bowl of porridge
	**The yoghurt, custard, quark filled with:**
__________	Cornflakes
__________	Muesli
__________	Cruesli
__________	Fruit, namely ……………………………………………………….…………………
__________	Other, namely …………………………………………………………………………
	**Fruit**
__________	Pieces of fruit, namely …………………………………………………………….
Remarks:	If you ate/drank something that is not listed above, you can enter it here.<br>……………………………………………………………………………………..……<br>………………………………………………………………………………………<br>………………………………………………………………………………………<br>………………………………………………………………………………………<br>………………………………………………………………………………………<br>………………………………………………………………………………………<br>………………………………………………………………………………………<br>………………………………………………………………………………………

* Cross-out what does not apply

Patient identification number

Food diary version 1.0

Day 2: During the day date:……………….. ………..day

Quantity	Food item
	**Drinks:**
__________	Water cup/mug/glass*
__________	Teacup/mug/glass*
__________	Coffee cup/mug/glass*
__________	*Skimmed/semi-skimmed/full fat milk cup/mug/glass**
__________	Buttermilk cup/mug/glass*
__________	*Skimmed/semi-skimmed/full fat chocolate milk cup/mug/glass**
__________	*Skimmed/semi-skimmed/full fat drinking yoghurt cup/mug/glass**
__________	Beer glass/can/bottle*
__________	Glass of wine
__________	Fruit juice namely…………………………………………..……cup/mug/glass*
__________	Soft drink namely………………………………………………cup/mug/glass*
__________	Other, namely ………………………………………………………………..
	**Cakes and confectionary**
__________	Slice(s) of large cookie/cake type
__________	Cookie(s)/Biscuit(s)
__________	Pastry, type…………………………………………………….………….….....
__________	Candy bar mars / nuts / other………………………………………..……......
__________	Piece(s) of chocolate milk / dark / white*
__________	Sweets, type…………………………………………………………………
	**Savoury:**
__________	Cubes of cheese, type………………………………………………………
__________	Piece of sausage, type………………………………………………………..
__________	Toast with ………………………………………………………….................
__________	Handful(s) of chips/nuts
__________	Other, namely ………………………………………………………………
	**Fruit**
__________	Pieces of fruit, namely ……………………………………………………
Remarks:	If you ate/drank something that is not listed above, you can enter it here.<br>…………………………………………………………………………..……<br>……………………………………………………………………………<br>……………………………………………………………………………<br>……………………………………………………………………………<br>……………………………………………………………………………

* Cross-out what does not apply

Patient identification number

Food diary version 1.0

<u>Day 2: HOT MEAL</u> date:……………….. ………..day

Quantity	Food item
	**Soup:**
___________	*Plate / cup* soup, packet / can / homemade**
	What is in it?
	………………………………………………………………………………………..
	………………………………………………………………………………………..
	**Vegetables / Salad:**
___________	Serving spoons vegetables, type………………………………………………………....
___________	Tablespoons applesauce
___________	Serving spoons raw vegetables, type……………………………………………...
	With sauce / dressing* type……………………………………………………..
	………………………………………………………………………………………..
	**Meat / meat substitutes:**
___________	Gram beef, type…………………………………………………………………
___________	Gram pork, type…………………………………………………………………
___________	Gram poultry, type………………………………………………………………..
___________	Gram fish, type……………………………………………………………………
___________	Gram meat substitute, type……………………………………………………….
___________	Gram other, namely, ……………………………………………………………......
___________	Tablespoons *frying butter / olive oil / sunflower oil**
	**Potatoes / rice and pasta:**
___________	Piece of potato (the size of 1 egg) boiled / fried*
___________	Serving spoons fries
___________	Serving spoons cooked rice, noodles
___________	Serving spoons pasta (e.g. pasta, macaroni, noodles),
___________	Other, namely…………………………………………………………………………
	**Sauce:**
___________	Gravy spoons gravy, made from butter / margarine / cooking and frying fat *
___________	Tablespoons sauce, type..
	**One-pot meal:**
	If you ate rice or pasta as a one-pot meal today (e.g. nasi, bami, macaroni dish, stew), how did you prepare it?
	………………………………………………………………………………………..
	………………………………………………………………………………………..
	………………………………………………………………………………………..

Food diary version 1.0

	**Dessert:**
__________	Dish of low-fat/semi-skimmed/full-fat yoghurt
__________	Dish of low-fat/semi-skimmed/full-fat custard
__________	Dish of low-fat/semi-skimmed/full-fat quark
__________	Dish of pudding Ball/piece* of ice cream, type………………………………....
__________	Piece of fruit, type…………………………………………………………………
__________	Block of cheese, type.......................………………………………………..
__________	Other, namely………………………………………………………..........
	**Drinks:**
__________	Water cup/mug/glass*
__________	Teacup/mug/glass*
__________	Coffee cup/mug/glass*
__________	*Skimmed/semi-skimmed/full fat milk cup/mug/glass**
__________	Buttermilk cup/mug/glass*
__________	*Skimmed/semi-skimmed/full fat chocolate milk cup/mug/glass**
__________	*Skimmed/semi-skimmed/full fat drinking yoghurt cup/mug/glass**
__________	Beer glass/can/bottle*
__________	Glass of wine
__________	Fruit juice namely…………………………………………..…….cup/mug/glass*
__________	Soft drink namely…………………………………………………cup/mug/glass*
__________	Other, namely …………………………………………………………………..
Remarks:	If you ate/drank something that is not listed above, you can enter it here.<br>…………………………………………………………………………………..……<br>………………………………………………………………………………………<br>………………………………………………………………………………………<br>………………………………………………………………………………………<br>………………………………………………………………………………………

* Cross-out what does not apply

Patient identification number

Food diary version 1.0

Day 2: During the evening date:……………….. ………..day

Quantity	Food item
	**Drinks:**
__________	Water cup/mug/glass*
__________	Teacup/mug/glass*
__________	Coffee cup/mug/glass*
__________	*Skimmed/semi-skimmed/full fat milk cup/mug/glass**
__________	Buttermilk cup/mug/glass*
__________	*Skimmed/semi-skimmed/full fat chocolate milk cup/mug/glass**
__________	*Skimmed/semi-skimmed/full fat drinking yoghurt cup/mug/glass**
__________	Beer glass/can/bottle*
__________	Glass of wine
__________	Fruit juice namely……………………………………...……cup/mug/glass*
__________	Soft drink namely………………………………………………cup/mug/glass*
__________	Other, namely …………………………………………………………………...
	**Cakes and confectionary**
__________	Slice(s) of large cookie/cake type
__________	Cookie(s)/Biscuit(s)
__________	Pastry, type……………………………………………………….…………..................
__________	Candy bar mars / nuts / other…………………………………….……...................
__________	Piece(s) of chocolate milk / dark / white*
__________	Sweets, type……………………………………………………………………………
	**Savoury:**
__________	Cubes of cheese, type……………………………………………………………....
__________	Piece of sausage, type……………………………………………………….……..
__________	Toast with ………………………………………………………….............................
__________	Handful(s) of chips/nuts
__________	Other, namely …………………………………………………………………………
	**Fruit**
__________	Pieces of fruit, namely ………………………………………………………………
Remarks:	If you ate/drank something that is not listed above, you can enter it here.<br>……………………………………………………………………………..…………..<br>…………………………………………………………………………………………<br>…………………………………………………………………………………………<br>…………………………………………………………………………………………<br>………………………………………………………………………………………

* Cross-out what does not apply

Patient identification number

Food diary version 1.0

Day 3: Breakfast date:……………….. ………..day

Quantity	Food item
	**Bread:**
__________	Slice(s) white/brown/wholemeal bread
__________	Slice(s) rye bread
__________	Slice(s) currant bread
__________	Slice(s) sugar bread
__________	Piece(s) rolls
__________	Piece(s) soft buns
__________	Piece(s) croissant
__________	Piece(s) Other, namely …………………………………………………………....
	**Spread / topped with:**
__________	Butter
__________	Margarine, brand and type,…………………………………………………………
__________	Half-fat margarine, brand and type,……………………………………………...
	Cheese, type…………………………………………………………………………
__________	Cold cuts, type……………………………………………………………………...
__________	Sweet topping, type………………………………………………………………...
__________	Other, namely ……………………………………………………………….......
__________	
	**Yogurt**
__________	Bowl of low-fat/semi-skimmed/full-fat yoghurt
__________	**Custard**
__________	Bowl of low-fat/semi-skimmed/full-fat custard
	**Quark**
__________	Bowl of low-fat/semi-skimmed/full-fat quark
	**Porridge**
__________	Bowl of porridge
	**The yoghurt, custard, quark filled with:**
__________	Cornflakes
__________	Muesli
__________	Cruesli
__________	Fruit, namely ……………………………………………………………..………....
__________	Other, namely ……………………………………………………………………...
	**Fruit**
__________	Pieces of fruit, namely ………………………………………………………

Patient identification number

Food diary version 1.0

	**Drinks:**
__________	Water cup/mug/glass*
__________	Teacup/mug/glass*
__________	Coffee cup/mug/glass*
__________	*Skimmed/semi-skimmed/full fat milk cup/mug/glass**
__________	Buttermilk cup/mug/glass*
__________	*Skimmed/semi-skimmed/full fat chocolate milk cup/mug/glass**
__________	*Skimmed/semi-skimmed/full fat drinking yoghurt cup/mug/glass**
__________	Fruit juice namely…………………………………………..…….cup/mug/glass*
__________	Soft drink namely……………………………………………….cup/mug/glass*
__________	Other, namely ………………………………………………………………..
Remarks:	If you ate/drank something that is not listed above, you can enter it here.<br>……………………………………………………………………………...……<br>…………………………………………………………………………………<br>…………………………………………………………………………………<br>…………………………………………………………………………………<br>…………………………………………………………………………………<br>…………………………………………………………………………………<br>…………………………………………………………………………………

* Cross-out what does not apply

Patient identification number

Food diary version 1.0

Day 3: DURING THE MORNING date:……………….. ………..day

Quantity	Food item
__________	**Drinks:**
__________	Water cup/mug/glass*
__________	Teacup/mug/glass*
__________	Coffee cup/mug/glass*
__________	*Skimmed/semi-skimmed/full fat milk cup/mug/glass**
__________	Buttermilk cup/mug/glass*
__________	*Skimmed/semi-skimmed/full fat chocolate milk cup/mug/glass**
__________	*Skimmed/semi-skimmed/full fat drinking yoghurt cup/mug/glass**
__________	Fruit juice namely…………………………………………..…….cup/mug/glass*
__________	Soft drink namely………………………………………………cup/mug/glass*
__________	Other, namely ………………………………………………………………..
	**Cakes and confectionary**
__________	Slice(s) of large cookie/cake type
__________	Cookie(s)/Biscuit(s)
__________	Pastry, type…………………………………………………….…………..........
__________	Candy bar mars / nuts / other………………………………………….……...........
__________	Piece(s) of chocolate milk / dark / white*
__________	Sweets, type…………………………………………………………………………
	**Savoury:**
__________	Cubes of cheese, type……………………………………………………………
__________	Piece of sausage, type…………………………………………………………….
__________	Toast with ……………………………………………………………..................
__________	Handful(s) of chips/nuts
__________	Other, namely …………………………………………………………………….
	**Fruit**
__________	Pieces of fruit, namely ………………………………………………………………
Remarks:	If you ate/drank something that is not listed above, you can enter it here.<br>……………………………………………………………………………………..………<br>…………………………………………………………………………………………<br>…………………………………………………………………………………………<br>…………………………………………………………………………………………<br>…………………………………………………………………………………………<br>…………………………………………………………………………………………<br>…………………………………………………………………………………………

* Cross-out what does not apply

Patient identification number

Food diary version 1.0

Day 3: Bread meal date:……………….. ………..day

Quantity	Food item
	**Bread:**
__________	Slice(s) white/brown/wholemeal bread
__________	Slice(s) rye bread
__________	Slice(s) currant bread
__________	Slice(s) sugar bread
__________	Piece(s) rolls
__________	Piece(s) soft buns
__________	Piece(s) croissant
__________	Piece(s) Other, namely ………………………………………………………..
	**Spread / topped with:**
__________	Butter
__________	Margarine, brand and type,……………………………………………………
__________	Half-fat margarine, brand and type,…………………………………………
	Cheese,
__________	type………………………………………………………………………………..
__________	Cold cuts, type……………………………………………………………………
__________	Sweet topping, type………………………………………………………………
__________	Other, namely ………………………………………………………………...
	**Drinks:**
__________	Water cup/mug/glass*
__________	Teacup/mug/glass*
__________	Coffee cup/mug/glass*
__________	*Skimmed/semi-skimmed/full fat milk cup/mug/glass**
__________	Buttermilk cup/mug/glass*
__________	*Skimmed/semi-skimmed/full fat chocolate milk cup/mug/glass**
__________	*Skimmed/semi-skimmed/full fat drinking yoghurt cup/mug/glass**
__________	Fruit juice namely……………………………………...…….cup/mug/glass*
__________	Soft drink namely………………………………………………cup/mug/glass*
__________	Other, namely ………………………………………………………………...
	**Yogurt**
__________	Bowl of low-fat/semi-skimmed/full-fat yoghurt
	**Custard**
__________	Bowl of low-fat/semi-skimmed/full-fat custard
	**Quark**

Patient identification number

Food diary version 1.0

__________	Bowl of low-fat/semi-skimmed/full-fat quark
	**Porridge**
__________	Bowl of porridge
	**The yoghurt, custard, quark filled with:**
__________	Cornflakes
__________	Muesli
__________	Cruesli
__________	Fruit, namely ………………………………………………………..…………………
__________	Other, namely …………………………………………………………………………
	**Fruit**
__________	Pieces of fruit, namely …………………………………………………………….
Remarks:	If you ate/drank something that is not listed above, you can enter it here.<br>………………………………………………………………………………………..……<br>……………………………………………………………………………………………<br>……………………………………………………………………………………………<br>……………………………………………………………………………………………<br>……………………………………………………………………………………………<br>……………………………………………………………………………………………<br>……………………………………………………………………………………………<br>……………………………………………………………………………………………

* Cross-out what does not apply

Patient identification number

Food diary version 1.0

Day 3: During the day date:……………….. ………..day

Quantity	Food item
	**Drinks:**
________	Water cup/mug/glass*
________	Teacup/mug/glass*
________	Coffee cup/mug/glass*
________	*Skimmed/semi-skimmed/full fat milk cup/mug/glass**
________	Buttermilk cup/mug/glass*
________	*Skimmed/semi-skimmed/full fat chocolate milk cup/mug/glass**
________	*Skimmed/semi-skimmed/full fat drinking yoghurt cup/mug/glass**
________	Beer glass/can/bottle*
________	Glass of wine
________	Fruit juice namely………………………………………...…….cup/mug/glass*
________	Soft drink namely…………………………………………….cup/mug/glass*
________	Other, namely ………………………………………………………………..
	**Cakes and confectionary**
________	Slice(s) of large cookie/cake type
________	Cookie(s)/Biscuit(s)
________	Pastry, type…………………………………………………….…………….....
________	Candy bar mars / nuts / other……………………………………….…….......
________	Piece(s) of chocolate milk / dark / white*
________	Sweets, type…………………………………………………………………
	**Savoury:**
________	Cubes of cheese, type………………………………………………………
________	Piece of sausage, type………………………………………………………..
________	Toast with ……………………………………………………………................
________	Handful(s) of chips/nuts
________	Other, namely ………………………………………………………………
	**Fruit**
________	Pieces of fruit, namely ………………………………………………………
Remarks:	If you ate/drank something that is not listed above, you can enter it here.<br>……………………………………………………………………………..……<br>………………………………………………………………………………………<br>………………………………………………………………………………………<br>………………………………………………………………………………………<br>………………………………………………………………………………………

* Cross-out what does not apply

Patient identification number

Food diary version 1.0

Day 3: HOT MEAL date:……………….. ………..day

Quantity	Food item
	**Soup:**
___________	*Plate / cup* soup, packet / can / homemade**
	What is in it?
	………………………………………………………………………………....................
	....
	**Vegetables / Salad:**
___________	Serving spoons vegetables, type………………………………………………………....
___________	Tablespoons applesauce
___________	Serving spoons raw vegetables, type……………………………………………......
	With sauce / dressing* type……......…....…………………………….....................
	…………………………………………………………………………………………..
	**Meat / meat substitutes:**
___________	Gram beef, type……………………………………………………………………….
___________	Gram pork, type…...…………………………………………………………………….
___________	Gram poultry, type……………………………………………………………………
___________	Gram fish, type…………………………………………………………………………
___________	Gram meat substitute, type…………………………………...…………………
___________	Gram other, namely, …………………………………………………………..........
___________	Tablespoons *frying butter / olive oil / sunflower oil**
	**Potatoes / rice and pasta:**
___________	Piece of potato (the size of 1 egg) boiled / fried*
___________	Serving spoons fries
___________	Serving spoons cooked rice, noodles
___________	Serving spoons pasta (e.g. pasta, macaroni, noodles),
___________	Other, namely………………………………………………………………………..
	**Sauce:**
___________	Gravy spoons gravy, made from butter / margarine / cooking and frying fat *
___________	Tablespoons sauce, type...
	**One-pot meal:** If you ate rice or pasta as a one-pot meal today (e.g. nasi, bami, macaroni dish, stew), how did you prepare it? ……………………………………………………………………………..……… ……………………………………………………………………………….……… ……………………………………………………………………………………….

rijksuniversiteit groningen

umcg

Patient identification number

Food diary version 1.0

	**Dessert:**
__________	Dish of low-fat/semi-skimmed/full-fat yoghurt
__________	Dish of low-fat/semi-skimmed/full-fat custard
__________	Dish of low-fat/semi-skimmed/full-fat quark
__________	Dish of pudding Ball/piece* of ice cream, type………………………………....
__________	Piece of fruit, type…………………………………………………………………
__________	Block of cheese, type........................……………………………………..
__________	Other, namely………………………………………………………..........
	**Drinks:**
__________	Water cup/mug/glass*
__________	Teacup/mug/glass*
__________	Coffee cup/mug/glass*
__________	*Skimmed/semi-skimmed/full fat milk cup/mug/glass**
__________	Buttermilk cup/mug/glass*
__________	*Skimmed/semi-skimmed/full fat chocolate milk cup/mug/glass**
__________	*Skimmed/semi-skimmed/full fat drinking yoghurt cup/mug/glass**
__________	Beer glass/can/bottle*
__________	Glass of wine
__________	Fruit juice namely…………………………………………...…….cup/mug/glass*
__________	Soft drink namely…………………………………………………cup/mug/glass*
__________	Other, namely …………………………………………………………………
Remarks:	If you ate/drank something that is not listed above, you can enter it here.<br>……………………………………………………………………………...……<br>…………………………………………………………………………………<br>…………………………………………………………………………………<br>…………………………………………………………………………………<br>…………………………………………………………………………………

* Cross-out what does not apply

Patient identification number

Food diary version 1.0

Day 3: During the evening date:……………….. ………..day

Quantity	Food item
	**Drinks:**
__________	Water cup/mug/glass*
__________	Teacup/mug/glass*
__________	Coffee cup/mug/glass*
__________	*Skimmed/semi-skimmed/full fat milk cup/mug/glass**
__________	Buttermilk cup/mug/glass*
__________	*Skimmed/semi-skimmed/full fat chocolate milk cup/mug/glass**
__________	*Skimmed/semi-skimmed/full fat drinking yoghurt cup/mug/glass**
__________	Beer glass/can/bottle*
__________	Glass of wine
__________	Fruit juice namely……………………………………….…….cup/mug/glass*
__________	Soft drink namely………………………………………………cup/mug/glass*
__________	Other, namely …………………………………………………………………..
	**Cakes and confectionary**
__________	Slice(s) of large cookie/cake type
__________	Cookie(s)/Biscuit(s)
__________	Pastry, type…………………………………………………………….……………........
__________	Candy bar mars / nuts / other……………………………………….……...........
__________	Piece(s) of chocolate milk / dark / white*
__________	Sweets, type…………………………………………………………………………
	**Savoury:**
__________	Cubes of cheese, type…………………………………………………………………
__________	Piece of sausage, type………………………………………………………………..
__________	Toast with ………………………………………………………………..............
__________	Handful(s) of chips/nuts
__________	Other, namely ……………………………………………………………………….
	**Fruit**
__________	Pieces of fruit, namely ………………………………………………………………
Remarks:	If you ate/drank something that is not listed above, you can enter it here.<br>…………………………………………………………………………..………<br>…………………………………………………………………………………<br>…………………………………………………………………………………<br>…………………………………………………………………………………<br>…………………………………………………………………………………<br>…………………………………………………………………………………...

* Cross-out what does not apply
