# Supplementary material for: Relative Validity of the Groningen IBD Nutritional Questionnaire (GINQ-FFQ): A Food Frequency Questionnaire Designed to Assess Nutritional Intake in Patients with Inflammatory Bowel Disease
Source: Nutrients. 2025 Jan 10;17(2):239. doi: 10.3390/nu17020239 (PMC11768067; doi:10.3390/nu17020239)
Supplement: Supplementary file 1 [file nutrients-17-00239-s001.zip › Table S1 - List of foodgroups.pdf]

Supplementary table 1. Identification of foods

Main category	Food items	Products
Breakfast products	Breakfast drinks (readymade)+	
Breakfast grains	Breakfast cereals	Muesli
		Cruesli
		Wheat germ
		Bran
	Porridge grains	Oatmeal
		Brinta
		Semolina
	Processed grains	Cornflakes
		Rice crispies
Dairy	Milk+	Whole
		Semi-skimmed
		Skimmed
		Buttermilk
	Milk alternatives+	Lactose free
		Goat or sheep milk
		Soymilk
		Oatmilk
		Ricemilk
		Coconutmilk
		Almondmilk
	Chocolate milk	Whole
		Semi-skimmed
		Skimmed
		Oatmilk
		Soymilk
	Sweetened dairy drinks	Fruits
		Soy
		Other types of sweetened dairy drinks
	Health-improving dairy	Cholestrol lowering drinks
		Blood pressure lowering drinks
		Probiotic drinks
	Yogurt+	Whole
		Semi-skimmed
		Skimmed
		Soy
		Goat or sheep
		Sweetened/flavoured
		Fruits
	Quark+	Whole
		Semi-skimmed
		Skimmed
		Soy
		Goat or sheep
		Sweetened/flavoured
		Fruits
	Dutch custard	Whole
		Semi-skimmed
		Skimmed
		Soy
		Goat or sheep
		Sweetened/flavoured (vanille, chocolate, etc.)
		Fruits
	Ready-made porridge / pudding / dessert	Sweetened/flavoured
		Soy
	Homemade porridge / pudding	Custard
		Rice pudding
		Semolina pudding
	Sweetners added to yogurt / quark / etc	Sugar
		Syrup
		Honey
		Artifical sweetners
	Ice cream products	Milk-based
		Soy-based
		Water-based
		Milkshake
	Hard cheeses+	Soft-ripened
		Washed-rind
		Smear-ripened
		Blue
		Brined
		Processed
		Goat or sheep cheese
		Parmesan cheese
	Soft cheeses+	Dairy spreads
		Hüttekäse
		Cream cheese
		Foreign cheeses
		Goat or sheep cheese
		Processed cheese spread
		Mozarrella
		Mascarpone
		Feta
		Grated cheese

	Cream[+]	Sour cream
		Creme fraiche
		Cream (for cooking)
		Whipped cream (fresh)
		Whipped cream (spray can)
Bread and bread substitutes	Rusk / biscuit	White
		Whole grain
	Knäckebröd	Multigrain
	Breakfast crackers/crispbread	White
		Whole grain
		Multigrain
		Spelt
	Croissants	White
		Whole grain
		Spelt
		Cheese
		Ham/cheese
	Hard rolls/ rustic rolls[+]	White
		Brown
		Whole grain
		Multigrain
		Raisins / dried currants
		Bagels
		English muffin
		Remaining bread products
	Buns/rolls[+]	White
		Brown
		Whole grain
		Multigrain
		Raisins / dried currants
		Muesli
		Remaining bread products
	Bread slices/sandwich bread[+]	White
		Brown
		Whole grain
		Multigrain
		Spelt
		Raisins / dried currants
		Muesli
		Pumpernickel rye bread (light)
		Pumpernickel rye bread (dark)
		Sourdough
		Remaining bread products
Fats/oils	Butter spreads[+]	Butter
		Semi-skimmed butter
		Margarine (tub)
		Margarine (package)
		Diet margarine
		Margarine with plant sterols/-stanols
		Low-fat butter
		Diet low-fat butter
		Low-fat butter with plant sterols/-stanols
		Other types of spreads/butter
		No spread/butter
	Cooking fats/oils	Olive oil
		Peanut oil
		Sunflower oil
		Sesame oil
		Liquid margarine
		Baking and roasting products (solid)
		Baking and roasting products (liquid)
		Lard/bacon fat
		Spray can fats
Spreads	Nut pastas[+]	Peanut butter
		Nut paste (cashew, hazelnut, etc.)
		Sesame paste (tahin)
	Sweet spreads	Chocolate sprinkles or flakes
		Chocolate paste
		Hazelnut chocolate pasta
		Chocolate butter
		Chocolate slices
		Fruit sprinkles / forest fruit sprinkles
		Ground aniseed sprinkles / anise sprinkles ("gestampte muisjes" / "roze of blauwe muisjes")
		Jam/marmelade[+]
		Gingerbread sprinkles ("Schuddebuikjes")
		Anise sprinkles ("Anijshagelslag")
		Dutch Spiced Cookies Biscuits ("speculaas")
		Apple / pear syrup/jam
		Honey[+]
		Sugar[+]
	Savory (salad/humus) spreads[+]	Humus
		Marmite
		Sandwichspread
		Egg salad
		Chicken curry salad
		Pesto

		Tapenade
		Remaining type of salad / sandwichspread
Eggs	Fried eggs[+]	Fried eggs
		Omelet
	Boiled eggs[+]	Boiled
		Poached
Fruits	Dried fruits[+]	Cranberries
		Apricots
		Plums
		Apple
		Pears
		Dates
		Raisins
		Currants
		Tuttifrutti
		Figs
		Mango
		Banana
	Fresh fruits[+]	Strawberries
		Apricots
		Pineapple
		Apple
		Avocado
		Banana
		Berries
		Citrus fruit such as lemon, lime, orange, madarin, grapefruit
		Dates
		Grapes
		Raspberries
		Pomegranate
		Cherries
		Kiwi
		Coconut
		Mango
		Melon
		Nectarines
		Passion fruit
		Pear
		Peach
		Plums
		Figs
		Other types of fruit
	Processed fruits / Preserved fruits[+]	Apple sauce
		Cocktail fruit
		Prepackaged and sliced fruits / fruit salads
		Frozen fruits
Nuts, stone fruits, seeds	Nuts, stone fruits[+,°]	Almonds
		Cashew nuts
		Hazelnuts
		Macadamia nuts
		Brazil nuts
		Pecans
		Pistachio nuts
		Walnuts
		Chestnut
		Coconut
		Bar mix
	Peanuts[+,°]	Peanuts
	Seeds[+,°]	Pumpkin seeds
		Sesame seed
		Sunflower seeds
		Poppy seeds
		Pomegranate seeds
		Pine nuts
Meat and meat substitutes	Raw cold cuts[+]	Filet american
		Roast beef
		Carpaccio
	Processed cold cuts[+]	Boiled liver
		Liver sausage, pate, liver pate, liver cheese, berliner liver sausage
		Smoked meat ("rookvlees"), "fricandeau", gammon, chicken fillet, chicken roulade
		Bacon ("ontbijtspek", "katenspek")
		Cervelate, salami
		Sausage, roasted meat
		Grilled or cooked ham
		Other types of cold cuts
	Meat[+]	Beef
		Veal
		Pork
		Bacon
		Poultry
		Other types of meat
		Organ meat: liver, tong, kidneys, brains, sweetbread
		Game: gouze, moose, deer, hare, etc
		Lamb, goat, sheep, etc.
	Gravy	Meat gravy

		Prepackaged gravy / gravy powder
	Meat substitutes	Tofu
		Tempeh
		Seitan
		Quorn
		Valess
		Falafel
		Ready-made meat substitutes
Fish	Fresh fish+	Fatty fish
		White fish
	Raw fish+	Salty herring
		Sushi
		Caviar
	Processed fish+	Fried haddock
		Canned fish (tuna, salmon, sardines, etc.)
		Smoked fish (salmon, etc.)
		Vis sticks
	Shellfish and seafood+	Lobster
		Shrimps
		Crab
		Mussels
		Oysters
		Squid
		Escargot
		Seaweed
		Other types of shelfish and crustaceans
Vegetables	Vegetables+	Jerusalem artichoke
		Endive
		Artichoke
		Asparagus (green)
		Asparagus (white)
		Eggplant
		Celery
		Cauliflower
		Kale
		Spring onion/scallions
		Broccoli
		Mushrooms
		Cherry tomato
		Chinese cabbage
		Zucchini
		Garden peas
		Green cabbage
		Iceberg lettuce
		Cucumber
		Kohlrabi
		Cabbage lettuce
		Sea lavender/limonium
		Corn
		Pak Choy
		Bell pepper
		Pea pods
		Pumpkin
		Purslane
		Leeks
		Turnips
		Turnip stalks
		Rhubarb
		Radicchio
		Radish
		Rettich
		Beetroot
		Red cabbage
		Savoy
		Salsify
		Haricot
		Green bean
		Spinach
		Pointed cabbage
		Brussels sprouts
		Bean sprouts
		Tomato
		Broad bean
		Onion
		Lamb's lettuce
		Fennel
		Winter purslane
		Belgian endive/chicory
		White cabbage
		Carrots
		Samphire/sea beans
		Sweet potato
		Sauerkraut
	Raw vegetables+	Endive
		Pickle
		Avocado

		Celery
		Cauliflower
		Kale
		Cucumber with peel
		Cucumber without peel
		Cabbage types
		Paprika
		Radish
		Lettuce: iceberg
		Lettuce: head lettuce
		Lettuce: Arugula
		Lettuce: lamb's lettuce
		Spinach
		Bean sprouts
		Tomato
		Cress
		Onion
		Fennel
		Belgian endive/chicory
		Root
		Silverskin unions
Legumes	Legumes[+]	Brown, white, black beans
		Split peas
		Capuchin
		Green beans (borlotti)
		Lima beans
		Lentils
		Chickpeas
		Kidney beans
		Soybeans
Grain products	Pasta[+]	White
		Whole grain
		Stuffed pasta / home-made pasta
		Noodles/ramen
		Pasta salads
	Rice / other grains[+]	White
		Brown
		African
		Risotto
		Nut rice
		Couscous
		Bulgur
		Quinoa
		Cauliflower / broccoli
		Millet, barley
	Potatoes[+]	Boiled oder mashed
		Fries
		Baked/fried
		Potato slices, croquettes, rösti etc.
	Toast, bread, wraps, pita, baguette, etc[+]	Wraps, fajitas, tortilla's
		Taco's
		Turkish bread
		Nan bread
		Pita bread
		Baguette
Sauces	Warm sauces	Bechamel/white sauce
		Mushroom
		Hollandaise
		Cheese
		Satay
		Tomato
		Other types of warm sauces
	Dressing[+]	Oil-based dressing
		Mayonnaise-based dressing
		Yoghurt-based dressing
		Other types of dressing
	Red sauces[+]	Ketchup/curry
		Chilisauce
		BBQ sauce
	Mayonnaise and other non-red sauces[+]	Mayonnaise
		Fry sauce
		Garlic sauce
		Cocktail sauce
		Mosterd
		Soy sauce
		Fish sauce
		Wasabi
Additions to meals	Super foods[+]	Goji berries
		Dried mulberries
		Flax seeds
		Chia seeds
		Hemp seeds
	Seasoning[+]	Sweet soy sauce
		Sambal
		Shiracha
		Lemon juice

		Vinegar
		Balsamic vinegar
		Maggi seasoning sauce
		Mango chutney
		Mustard
		Dessicated coconut
		Shrimp paste (Terasi)
		Salt
		Nutritional yeast
		Tomato paste
		Instant bouillon cubes / powder
		Miso
	Spices & herbs[+]	Anise
		Basil
		Chives
		Chili pepper / cayenne pepper
		Lemongrass
		Dill
		Dragon
		Mace
		Ginger
		Cinnamon
		Cardamom
		Caraway
		Curry
		Chervil
		Garlic
		Cumin
		Coriander
		Clove
		Turmeric
		Laos
		Bay leaves
		Lovage
		Marjoram
		Spearnint
		Nutmeg
		Oregano
		Paprika
		Pepper
		Parsley
		Rosemary
		Saffron
		Sage
		Celery
		Thyme
		Cress
		Onion
		Vanilla
	Spiciness	Midly spiced
		Extremely spiced
	Olives / bell peppers[+]	Black olives
		Green olives
		Pappadew
		Grilled bell pepers
Fast food	Fast food[+]	Chinese/Indian take out
		Burgers/fries take out
		Pizza take out, frozen, home-made
		Tacos/burritos take out
		Donor / shawarma / gyros take out
		Noodles take out
		Asian streetfood take out
		Other types of take out food
		Ready made meals (bought in supermarket)
		Pancakes
		"Poffertjes"
Soup	Soup[+]	Cream soup
		Bouillon
		Instant soup
Savory snacks	Chips / pretzels	Potato-based
		Rice-based
		Popcorn[°]
		Vegetable-based
	Fried snacks	"Kroket" (croquette)
		"Bitterballen" (round croquettes)
		"Eierbal" (round croquette with a boiled egg in the middle)
		"Frikandel" (minced meat hot dog)
		"Kaassoufflé" (cheese souffle)
		"Saucijzenbroodje"/"worstenbroodje" (sausge roll pastries)
		Other types of fried snacks
Sweet snacks	Chewing gum	With sugar
		Without sugar
	Licorice	Salt
		Sweet
	Sweets/candy	Winegums, etc.
	Bonbon	With nuts

		With alcohol
	Chocolate	With/without nuts
		Dark, milk, white
	Candy bars	Celebrations, normal, king-size
	Gingerbread[+] / cookie bars / nutritional biscuits	Normal gingerbread
		Whole grain gingerbread
		White sugar candy gingerbread
		Raisins gingerbread
		Nuts gingerbread
		Sweetened flavors gingerbread: apple, caramel, ginger, sea salt-caramel, chocolate, etc.
		"Captein koek"
		Liga
		Sultana
		Muesli bars
		Protein bars
	Cake / large biscuits	Cake
		Large (filled) cookies
		Doughnuts
		Brownies
	Small cookies / biscuits	Biscuits, cookies, "speculaas", "pepernoten", etc.
	Pastry / pie	Pastry
		Pie
Beverages	Fruit juice	Prepackaged fruit juice
		Fresh/smoothie
		Slow-juice
		Apple
		Pear
		Orange
		Grapefruit
		Mango
		Berries
		Multivitamin
	Vegetable juice	Prepackaged vegetable juice
		Fresh/smoothie
		Slow-juice
		Tomato
		Carrot
		Beetroot
		Pickle
	Tap water	
	Bottled water / sparkled water	
	Coffee[-]	Caffeinated/decaffeinated
		Coffee, cappuccino, esspresso
		Fresh, filter, instant
	Tea	Black
		Green
		Herbs
		Fruit
	Coffee milk	Whole
		Semi-skimmed
		Skimmed
		Soy, oats, almond, rice, etc.
		Whitner/creamer
	Sweeteners[+]	Sugar
		Honey
		Agave/maple syrup
		Artificial sweetners
	Soda[-]	Carbonated, non-carbonated
		Iced tea
	Lemonade	Syrup lemonade
	Sport drinks	
	Energy drinks	
	Protein shakes	
	Alcoholic beverages	Beer
		Wine
		Fortified wines
		Spiritis
		Mixed drinks
		Other types of alcoholic drinks
		Non-alcoholic drinks

[+] *Added to meals (breakfast, lunch, dinner) (or beverages) or eaten as snack in between meals*

[°] *Salted or unsalted*

[-] *Caffeinated or decaffeinated*
